# Acupuncture for tumor-related depression: a systematic review and meta-analysis

**DOI:** 10.3389/fonc.2023.1198286

**Published:** 2023-08-08

**Authors:** Xiaoyi Li, Yang Wang, Liu Wu, Xinyu Zhao, Tianmin Zhu

**Affiliations:** ^1^ School of Acupuncture and Tuina, Chengdu University of Traditional Chinese Medicine, Chengdu, China; ^2^ School of Sports Medicine and Health, Chengdu Sport University, Chengdu, China; ^3^ School of Rehabilitation and Health Preservation, Chengdu University of Traditional Chinese Medicine, Chengdu, China

**Keywords:** acupuncture, tumor, depression, systematic review, meta-analysis

## Abstract

**Introduction:**

Tumor-related depression is a series of symptoms or states triggered by a tumor as the basic disease. It does not belong to psychiatric depression but dramatically affects individuals’ quality of life. Acupuncture is extensively used to treat tumor-related depression, but the effect of body acupuncture on tumor-related depression is still unsubstantiated. This work, therefore, set out to assess the effect of acupuncture on tumor-related depression.

**Methods:**

Eight databases were searched from inception to October 2022 for randomized controlled trials (RCTs). Two researchers separately implemented the database search, study selection, data extraction, and quality assessment. All analyses were performed by using Review Manager 5.3.

**Results:**

A total of 10 studies, including 725 participants, were included. A majority of studies recruited patients diagnosed with various tumor types and statuses. Meta-analysis revealed that acupuncture had a beneficial effect compared with usual care on the Hamilton depression scale (HAMD) (mean difference (MD) = −2.23, 95% CI [−4.43, −0.03], *p* = 0.05), self-rating depression scale (SDS) (MD= −6.22, 95% CI [−10.67, −1.78], *p* = 0.006), effective rate (RR = 1.23, 95% CI [1.06, 1.43], *p* = 0.006), and quality-of-life questionnaire (QLQ-C30) (MD = 6.08, 95% CI [3.72, 8.43], *p*<0.0001). In the dimension of the HAMD (MD = −4.41, 95% CI [−6.77, −2.05], *p* = 0.0002) and SDS (MD = −9.19, 95% CI [−13.14, −5.24], *p* <0.00001), subgroup analysis also highlighted that acupuncture combined with usual care had an advantage over usual care. However, there was no superiority in acupuncture itself compared to usual care on the HAMD (MD = −1.25, 95% CI [−4.34, −1.84], *p* = 0.43) and SDS (MD = −3.08, 95% CI [−11.14, 4.98], *p* = 0.45). Acupuncture also reduced the incidence of adverse effects (RR=0.43, 95% CI [0.23, 0.80], *p* = 0.008).

**Conclusion:**

Acupuncture is a safe and effective complementary therapy for tumor-related depression. This technique can provide clinical references for the medical field.

**Systematic review registration:**

https://www.crd.york.ac.uk/PROSPERO, identifier CRD42022372513.

## 1 Introduction

Tumor-related depression is a kind of pathological state or syndrome associated with a tumor. The main symptoms comprised poor mentation, loss of interest, lack of energy, pessimism, self-guilt and suicidal tendencies (1). The relationship between cancer and depression is a comorbidity in the process; they affect each other (2). Tumors may increase susceptibility to depression in patients. The pressure of their deteriorating physical condition, chemotherapy, and surgery may contribute to the occurrence and progression of depression (3, 4). Meanwhile, psychological problems like depression probably had a bearing on the high mortality and low survival rate of tumor patients (5). Depression is common in tumor individuals, with the highest incidence of accompanied pathological emotions (6). The prevalence of tumor-related depression ranged from 17.5% to 95.3% in China and 12.5% to 33.4% in foreign countries (7). This disorder can induce severe consequences, including hazarding someone’s quality of life, resisting anti-tumor therapy, shortening survival, and even destroying one’s family (8). Therefore, depressive disorders resulting from tumors have attracted growing concern in the medical community.

The etiology of tumor-related depression is the consequence of multiple factors. Psychological factors run through the whole process of tumor occurrence, diagnosis, and treatment (1). The pathogenesis of tumor-related depression is still unclear. At present, the hypotheses of abnormal neurotransmitter metabolism, chronic inflammatory mechanisms, and imbalance of hypothalamic-pituitary-adrenal (HPA) axis regulation have been highly accepted (3, 9, 10). In the current stage, how to accurately diagnose, timely prevent, and properly cure tumor-related depression remains a challenge.

Generally, there are pharmacological and nonpharmacological approaches to treating tumor-related depression. A therapeutic protocol mainly based on antidepressants may result in headaches, fluctuation of blood pressure, arrhythmias, and impairment of liver and kidney function (11). As a characteristic therapy of traditional Chinese medicine, acupuncture belongs to a nonpharmacological approach that has been widely applied in palliative cancer care (12). The advantages of acupuncture consist of safety, rare and mild side effects, and high patient acceptance. Its status in the treatment of tumor-related depression is rising. In 2014 and 2017, the Society for Integrative Oncology (SIO) released clinical practice guidelines to announce that acupuncture can alleviate depressive disorders in breast cancer patients (13, 14). A retrospective study described that acupuncture was as effective as medication for cancer-related depression (15). Likewise, randomized controlled trials (RCTs) have validated the efficacy and safety of acupuncture for such patients (16, 17). However, there were still some studies that argued that acupuncture failed to improve the relevant scale of tumor-related depression better than usual care, like HAMD or SDS (18–20). Accordingly, the effect of acupuncture on tumor-related depression was controversial. An updated review is imperative to investigate associated evidence.

Currently, systematic review studies about acupuncture in palliating tumor-related symptoms are increasing (21–23). Only one review assessed the effect of acupuncture and acupressure on tumor-related depression (15). However, the acupressure points it included were mainly auricular points rather than body acupuncture. Accordingly, we conducted a systematic review and meta-analysis of existing RCTs to provide a clinical reference.

## 2 Methods

### 2.1 Study registration

This systematic review and meta-analysis are registered on PROSPERO (No. CRD42022372513).

### 2.2 Database and search strategy

The study was performed according to the Cochrane Handbook for Systematic Reviews of Interventions and followed the Preferred Reporting Items for Systematic Reviews and Meta-Analyses Statement (PRISMA) guidelines. The research data in this review were drawn from eight databases: PubMed, EMBASE, Web of Science, the Cochrane Library, Chinese Biomedical Literature Database (CBM), China National Knowledge Infrastructure (CNKI), Wanfang Database, and VIP Database. RCTs published from inception to October 2022 were searched. There was no limitation on the language.

The search terms used were as follows: (“acupuncture” OR “electroacupuncture” OR “needl*”) AND (“depressive symptoms” OR “emotional depression” OR “depression”) AND (“cancer” OR “benign neoplasm*” OR “malignancy” OR “malignant neoplasm*” OR “neoplas*” OR “tumor*” OR “neoplasms”).

### 2.3 Inclusion and exclusion criteria

#### 2.3.1 Study type

Only RCTs were applicable. Conference papers, guidelines, reviews, and republished literature were excluded.

#### 2.3.2 Participants

Patients diagnosed with tumor-related depression, regardless of tumor stage, location, or pathological type were included.

#### 2.3.3 Interventions and comparisons

Acupuncture (including body acupuncture and electroacupuncture) used as an intervention to treat tumor-related depression was covered. Trials that compared acupuncture plus usual care (medication, decoction, sham acupuncture, waitlisting, or any other recognized means) with usual care alone were also available. The trial was eligible as long as the control group adopted the same combined treatment as the observation group. Comparisons of different acupuncture techniques were excluded, such as scalp acupuncture, auricular acupuncture, laser acupuncture, or acupressure.

#### 2.3.4 Outcomes

The primary outcome measures contained the Hamilton depression scale (HAMD) and self-rating depression scale (SDS). At least one outcome indicator was described. To evaluate tumor patients’ general health, we used a quality-of-life questionnaire (QLQ-C30), which was established by the European Cancer Research and Treatment Institution. It was designed exclusively for tumor patients. In addition, the effective rate and adverse effects were evaluated. The HAMD reduced rate was used as the effective rate to assess the therapeutic effectiveness of tumor-related depression. HAMD reduced rate (%) = (score before treatment-score after treatment)/score before treatment × 100%, cure rate (reduced rate > 75%), effective rate (reduced rate 50%–75%), improved rate (reduced rate 25%–49%), and invalid rate (reduced rate < 25%). The effective rate = (total number of cases − invalid number of cases)/total number of cases × 100%.

### 2.4 Study selection and data extraction

Two authors worked independently on the selection and extraction processes, and disagreements were settled by discussion with a third author. First, in light of predetermined inclusion and exclusion criteria, all of the titles and abstracts were screened. Full-text articles were then obtained for further assessment. Data extraction incorporated basic information about the article (the first author, publication year, country, sample size), general materials about patients (age, course, type of tumor, and current anti-tumor therapy), traits of intervention and control groups (retention, frequency, course), outcomes, and adverse events.

### 2.5 Risk of bias assessment

Risk of bias assessments were performed with the Cochrane risk of bias tool (24). Two researchers separately evaluated the following domains: random sequence generation, allocation concealment, blinding of participants and personnel, blinding of outcome assessment, incomplete outcome data, selective reporting, and other bias. Each sphere was rated as having a low, high, or unclear risk of bias.

### 2.6 Data analysis

The meta-analysis was tackled by Review Manager version 5.3 software. HAMD, SDS, and QLQ-C30 scores belonging to continuous data are expressed as mean difference (MD) with a 95% confidence interval (CI). Effective rate and adverse effects belonging to dichotomous data are represented as risk ratios (RR) with 95% CI. We first assessed heterogeneity, and then subgroup analysis was conducted for the results with high heterogeneity. The selection of a fixed-effect model (*p* ≥ 0.1 and *I* ^2^ ≤ 50%) or a random-effect model (*p* < 0.1 and *I* ^2^ > 50%) was determined by the values of *p* and *I* ^2^. In addition, a sensitivity analysis was implemented to test the stability of the results. If necessary, publication bias analysis was depicted by a funnel plot.

## 3 Results

### 3.1 Study identification

**Figure 1:**
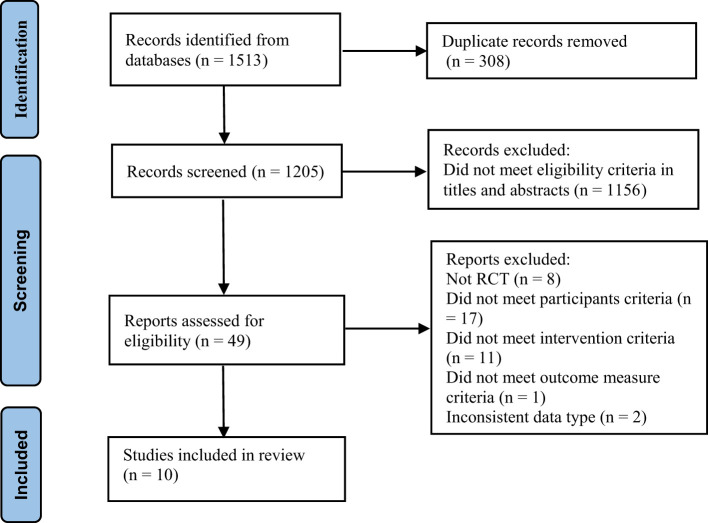
Study screening flow chart.

A total of 1,513 studies were searched from databases, of which 308 duplicate literature were removed and 1,156 publications were excluded for not conforming to the inclusion criteria according to the title and abstract. After examining the whole text, 10 articles were accessible for analysis (**Figure 1**; **Table 1**).

**Table 1:** Characteristics of the included RCTs for patients of tumor-related depression.

Author (year; country)	Sample Size (F/M); Mean Age (±SD)	Tumor type; tumor stage	Current anti-tumor treatment	Intervention (acupoints; retention; frequency; course)	Control	Outcomes	Adverse events
Deng (16) (2019; China)	AG: 30 (11/19) CG: 30 (10/20); AG: 53 ± 9 CG: 49 ± 11	Various cancers; III to IV	Chemotherapy	AT (LI4, PC6, LR3, HT7; 30 min; 2 times weekly; 4 weeks); UC	UC	HAMD; QLQ-C30	None
Lian (17) (2019; China)	AG: 60 (31/29) CG: 60 (32/28);AG: 62 ± 5 CG: 62 ± 6	Various cancers; various stages	Surgery, chemotherapy, radiotherapy, endocrinotherapy, biotherapy, targeted therapy	AT (CV12, GV20, GV24, BL15, BL18, BL20, HT7, LR3, KI3; 20 min; 5 times weekly; 6 weeks); UC	UC	HAMD; SDS	None
Pei (18) (2010; China)	AG: 31 CG: 36 AG: 51.76±10.21;CG: 48.34±8.79	Breast cancer; not reported	Not reported	AT (BL13, BL15, BL17, BL18, BL20, BL23; 30 min; 5 times weekly; 8 weeks)	UC	HAMD; SDS	3 in AG: palpitation (1), dizziness (1), 14 in CG: dizziness (3), nausea (4), perspiration (2), dry mouth (5)
Deng (19) (2018; China)	AG: 30 (12/18) CG: 30 (16/14); AG: 63.80±5.47 CG: 63.60 ±4.26	Various cancers; III to IV	Not reported	EAT (GB34, ST40, LI4, PC6, LR3, SP6, GV24^+^, GV20; 30 min; 2 times weekly; 4 weeks); UC	UC	HAMD; QLQ-C30	Not reported
Li (20) (2019; China)	AG: 30 CG: 28;AG: 52.37± 8.19 CG: 51.75±9.27	Breast cancer; I to III	Not reported	AT (GV20, GV24^+^, CV6, CV4, CV12, CV10, PC6, KI6, LI4, LR3; 30 min; 3 times weekly in the first 4 weeks, 2 times weekly in the last 4 weeks; 8 weeks)	UC	Effective rate; HAMD	8 in AG: tingling (6), pain (2); 15 in CG: digestive symptoms (12), fatigue (3)
Feng (25) (2011; China)	AG: 40 (26/14) CG: 40 (27/13); AG: 63.80±5.47 CG: 63.60±4.26	Various cancers, not reported	Not reported	AT (ST40, SP9, SP10, SP6, GV24^+^, GV20, EX-HN1, PC6, HT7; 20–30 min; daily; 30 days)	UC	Effective rate; HAMD; SDS	Not reported
Chen (26) (2012; China)	AG: 30 (18/12) CG: 30 (17/13); AG: 59.60±10.67 CG: 58.50±12.77	Various cancers, I to IV	Not reported	AT (BL13, BL15, BL18, BL20, BL23, GV23, LI11, PC5, BL62; 30 min; not reported); UC	UC	HAMD	Not reported
Liu (27) (2019; China)	AG: 40 (24/16) CG: 40 (20/20); AG: 63±13 CG: 63±12	Not reported, II to III	Not reported	AT (LI4, LR3, GV24^+^, GV20; 30 min; 5 times weekly; 3 months); UC	UC	Effective rate; HAMD	16 in AG: subcutaneous congestion (8), dizziness (5), headache (3); CG: not reported
Xia (28) (2019; China)	AG: 30 CG: 30; Not reported	Various cancers, not reported	On-treatment (no details)	EAT (GV20, GV24^+^, EX-HN1, 30 min; 5 times weekly; 4 weeks); UC	UC	HAMD	Not reported
He (29) (2022; China)	AG: 40 (21/19) CG: 40 (18/22); AG: 49.2±5.1 CG: 52.6±4.7	Various cancers, not reported	Not reported	AT (LI4, LR3, GV20, HT7, GV24^+^, EX-HN1, SP6, ST36, Ashi point; 20 min; daily; 4 weeks); UC	UC	SDS; QLQ-C30	Not reported

F, female; M, male; AG, acupuncture group; CG, control group; AT, acupuncture; UC, usual care; EAT, electroacupuncture; SD, standard deviation.

### 3.2 Characteristics of included studies

The numbers of participants ranged from 58 to 120, and a total of 725 patients were included, with 361 in the acupuncture group and 364 in the control group. It was reported that two participants dropped out of the investigation owing to long distances and intense needling sensations (20). Among the 10 studies, two consisted of breast cancer (18, 20), and the rest incorporated various types of neoplasms. The distribution of multiple tumors in the 725 samples was as follows: 179 breast cancer, 107 gastrointestinal tumors (gastric cancer, intestinal cancer, esophagus cancer, anal cancer); 112 lung cancer; 75 gynecological tumors (cervical cancer, fallopian tube tumor, endometrial cancer, ovarian cancer, vulvar cancer); 36 liver cancer; 13 nasopharyngeal carcinoma; 11 prostate cancer; 9 lymphoma; 6 testicular cancer; 6 thyroid cancer; 4 pancreatic cancer; and 167 other tumors. All of the studies performed in China and tumor-related depression is common in middle-aged and elderly people. Five trials specified the tumor stage (16, 19, 20, 26, 27). The participants were on anti-tumor treatment in three trials (16, 17, 28). The anti-tumor therapeutic schemes mainly consisted of chemotherapy, surgery, chemotherapy, radiotherapy, endocrinotherapy, biotherapy, and targeted therapy. Of all the included studies, three compared acupuncture with usual care (18, 20, 25), and the others compared the combination of acupuncture and usual care with usual care. A host of RCTs used manual acupuncture in the experimental group, except for two articles that used electroacupuncture (19, 28). The usual care primarily covered conventional antidepressants. The top acupoints used for tumor-related depression were GV20, GV24^+^, LR3, LI4, PC6, and HT7. Patients in the studies received acupuncture from twice a week to once a day for 4 to 12 weeks, each session varied from 20 to 40 min. Moreover, it took at least one month for acupuncture to exert effects on tumor-related depression. Three studies chose the HAMD and SDS as the depression measurement (17, 18, 25), six studies assessed depression with the HAMD alone (16, 19, 20, 26–28), and one study used the SDS alone (29). QLQ-C30 was implemented in three trials (16, 19, 29). Three studies reported effective rates (20, 25, 27). Three studies (18, 20, 27) reported side effects (pain, bleeding, dizziness, headache, etc.) resulting from acupuncture and other symptoms (nausea, perspiration, dry mouth, etc.) triggered by antidepressant drugs. The adverse events were slight, and medical interventions were dispensable. There were no adverse events in the two articles (16, 17), and the remaining studies did not mention relevant information.

### 3.3 Risk of bias assessment

**Figure 2:**
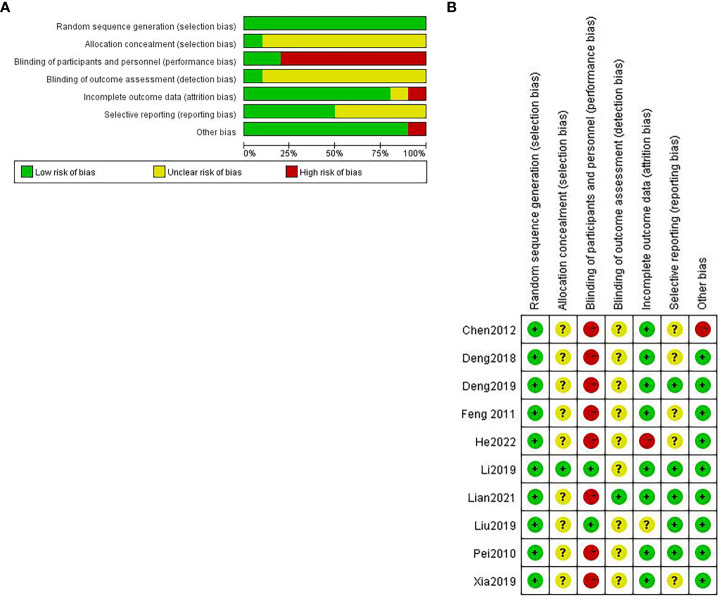
Assessment of risk of bias: **(A)** Risk of bias graph; **(B)** risk of bias summary.

The major accounts of the risk of bias were relevant to the blinding of participants and personnel. The risk of bias in the randomization process was quite low (**Figure 2A**).

For random sequence generation, five studies (16, 18, 25, 27, 29) used a random digit table; two studies took advantage of a computer to generate random numbers (17, 20); and the remaining did not point out techniques in details but generated randomly (19, 26, 28); all of them were considered to have a low risk of bias. For allocation concealment, one study (20) had a low risk of bias, and the others were regarded as having an unclear risk of bias. With regard to the blinding of participants and personnel, only two trials (20, 27) had a low risk of bias due to the specificity of acupuncture therapy. For blinding of outcome assessment, there was one article (17) having a low risk of bias; the rest were assessed as an unclear risk of bias. As for incomplete outcome data, one study (29) was deemed to have a high risk of bias because the safety indexes were absent, which had been mentioned in outcome measures. Another one (27) only illustrated the adverse events of the observation group but not the reported details of the control group. We cannot be certain about the data integrity; consequently, it is considered an unclear risk of bias. For selective reporting, five trials (19, 25, 26, 28, 29) had an unclear risk of bias as they did not report side effects; all the other trials had a low risk of bias. In light of other biases, one trial (26) did not explain the frequency of acupuncture. We cannot tell whether the observation group has the same frequency as the control group; thus, it has an unclear risk of bias (**Figure 2B**).

### 3.4 Data analysis

#### 3.4.1 HAMD

**Figure 3:**
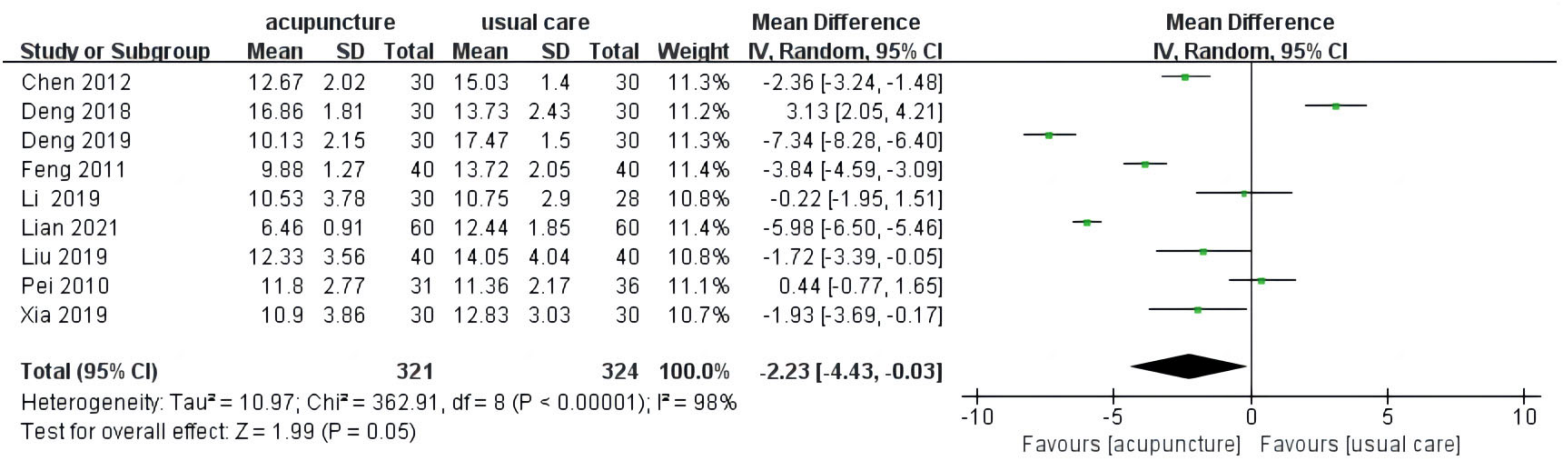
Forest plot of acupuncture vs. usual care on HAMD.

HAMD was considered an outcome indicator in nine studies (16–20, 25–28). Three results indicated that the improvement of HAMD score in the acupuncture group cannot be superior to usual care (18–20). There was high heterogeneity among these studies (*I* ^2 = ^98%, *p* < 0.00001). The pooled results of nine trials noted that there were more positive effects of acupuncture compared to usual care on HAMD score (MD = −2.23, 95% CI [−4.43, −0.03], *p* = 0.05; **Figure 3**).

#### 3.4.2 SDS

**Figure 4:**
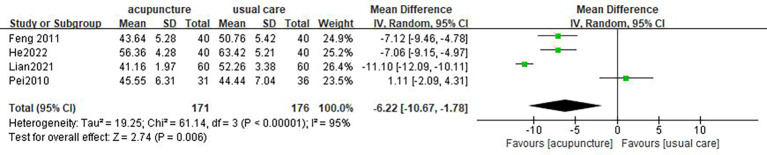
Forest plot of acupuncture vs. usual care on SDS.

Four studies (17, 18, 25, 29) used SDS as outcome measures. One study showed that there was no statistical difference in the improvement of SDS score between the acupuncture group and the usual care group (18). Great heterogeneity was revealed among the studies (*I* ^2 = ^95%, *p* < 0.00001), so a random-effect model was performed. The result of the meta-analysis showed that acupuncture was more efficient in decreasing SDS score than usual care (MD = −6.22, 95% CI [−10.67, −1.78], *p* = 0.006; **Figure 4**).

#### 3.4.3 Effective rate

**Figure 5:**
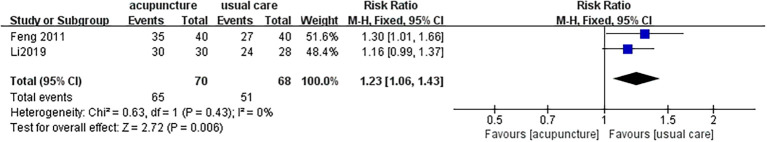
Forest plot of acupuncture vs. usual care on effective rate.

There were three studies that described the effective rate (20, 25, 27). Acupuncture plus usual care was applied to the treatment group in one study (27), and the result indicated that acupuncture plus usual care had a superior effect on tumor-related depression compared to just usual care. The other two studies (20, 25) only used acupuncture in the treatment group. The interventions were different, so the results of two studies (20, 25) were synthesized to assess the efficacy of acupuncture for tumor-related depression patients. The heterogeneity of the two studies was low (*I* ^2 = ^0%, *p* = 0.43). Every study proved the favorable effects of acupuncture for tumor-related depression compared to usual care. The pooled results suggested that acupuncture had a significantly better effective rate than usual care (RR = 1.23, 95% CI [1.06, 1.43], *p* = 0.006; **Figure 5**).

#### 3.4.4 QLQ-C30

**Figure 6:**
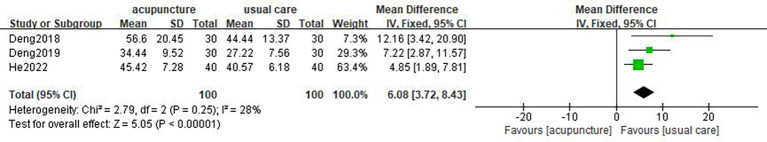
Forest plot of acupuncture vs. usual care on QLQ-C30.

There were three trials (16, 19, 29) applying QLQ-C30 as a tumor-related depression scale. We chose the quality-of-life (QL) domain to assess the general health of patients. Low heterogeneity was found among these studies (*I* ^2 = ^28%, *p* = 0.25). Meta-analysis validated that acupuncture can boost the QLQ-C30 score better than usual care (MD = 6.08, 95% CI [3.72, 8.43], *p* < 0.00001; **Figure 6**).

#### 3.4.5 Adverse effect

**Figure 7:**
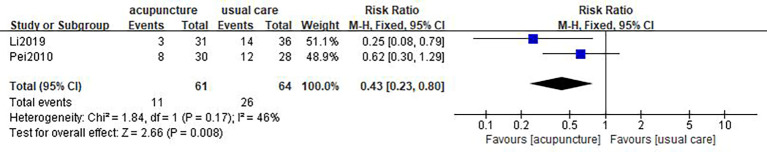
Forest plot of acupuncture vs. usual care on adverse effects.

Five studies recorded adverse events (16–18, 20, 27), but one study (27) was excluded from the meta-analysis due to incomplete data. Two studies showed no adverse events in two groups (16, 17), so we analyzed the consequences of two studies [18, 20]. There was low heterogeneity in these trials (*I* ^2 = ^46%, *p* = 0.17). The analysis suggested that acupuncture possessed superiority in reducing the incidence of adverse events (RR = 0.43, 95% CI [0.23, 0.80], *p* = 0.008; **Figure 7**).

#### 3.4.6 Subgroup analysis

**Figure 8:**
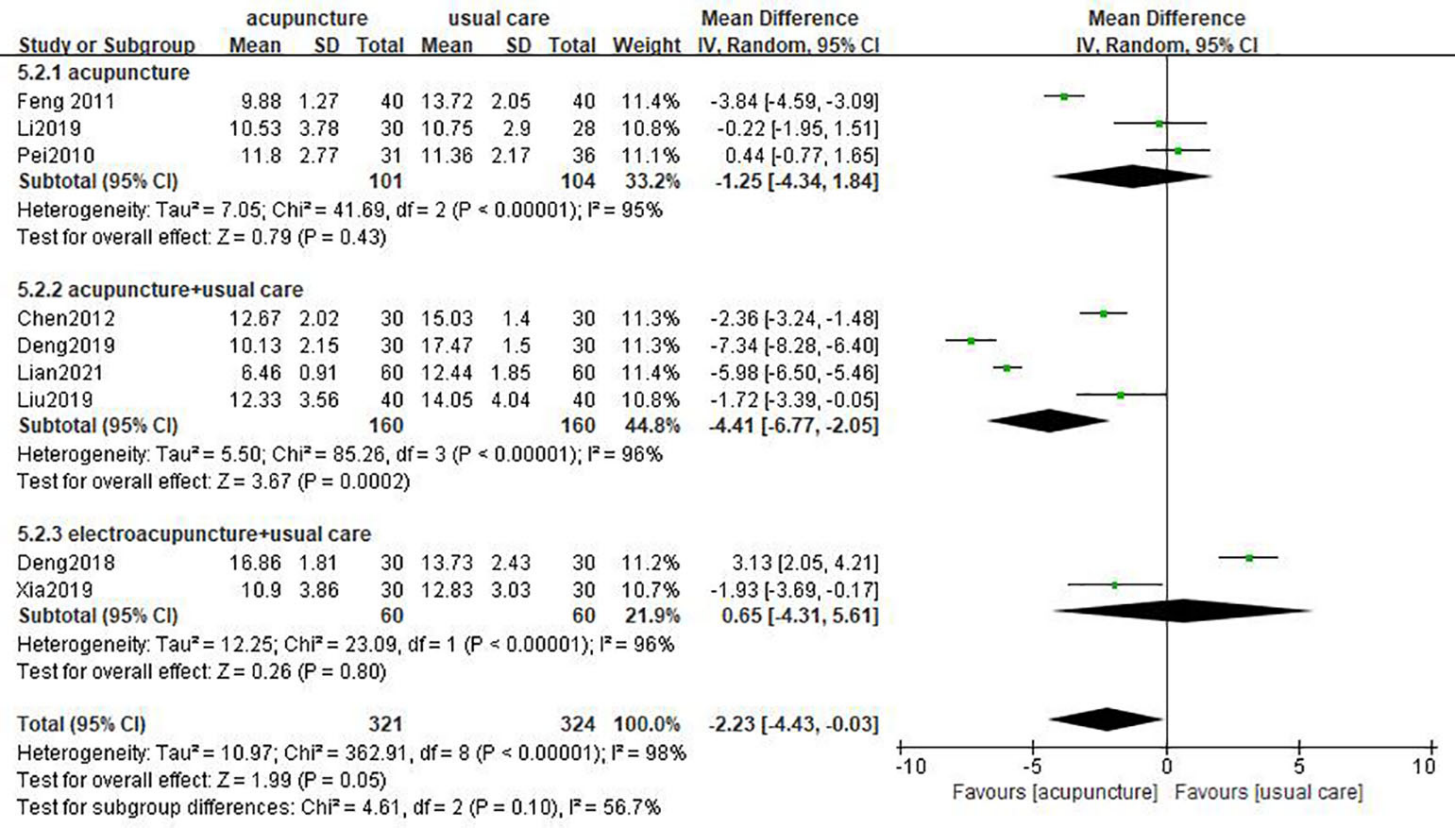
Subgroup analysis of different interventions on HAMD.

**Figure 9:**
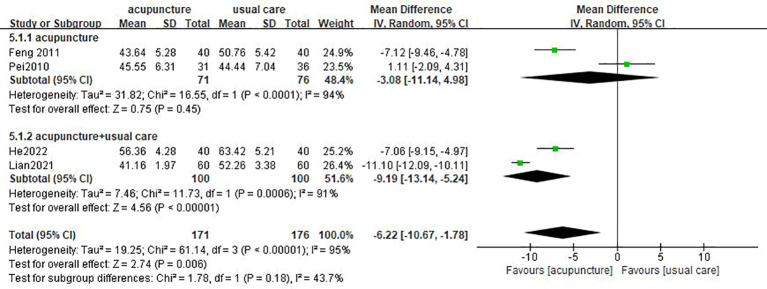
Subgroup analysis of different interventions on SDS.

According to different interventions, subgroup analysis was conducted on the HAMD score and SDS score. The merged results illustrated that acupuncture plus usual care can decrease the HAMD score better than usual care (MD= −4.41, 95% CI [−6.77, −2.05], *p* = 0.0002); whereas, acupuncture failed to have better improvement in HAMD score than usual care (MD = −1.25, 95% CI [−4.34, 1.84], *p* = 0.43). The result is applicable to electroacupuncture (MD = 0.65, 95% CI [−4.31, 5.61], *p* = 0.8; **Figure 8**). The results showed that acupuncture combined with usual care was more effective in decreasing the SDS score (MD = −9.19, 95% CI [−13.14, −5.24], *p* < 0.00001). However, there was no superior effect of acupuncture compared to usual care (MD = −3.08, 95% CI [−11.14, 4.98], *p* = 0.45; **Figure 9**).

#### 3.4.7 Sensitivity analysis

A sensitivity analysis was carried out for each data if the result did not reverse after removing any study, indicating that the result was reliable and stable. There was no statistical significance in the SDS score between two groups in one study, which made the result different from other data (18). For the HAMD score, except for three studies (18–20), the result would be changed because the three studies failed to exhibit that acupuncture has a superior effect on the HAMD score than usual care. In summary, the effect of acupuncture on HAMD and SDS scores should be treated cautiously. It is necessary to verify it through further studies. The remaining results were reliable and stable without changes.

## 4 Discussion

Patients with tumors were prescribed drugs in massive quantities; they would probably like to try nonpharmaceutical therapy. Acupuncture is a good choice. A previous study had implied that acupuncture may ameliorate emotional depression by strengthening hippocampal neuroplasticity and attenuating inflammation in the brain (30). One experiment found that acupuncture prevents the occurrence of depression by regulating the HPA axis (31). Additionally, acupuncture can regulate the level of neurotransmitters such as norepinephrine (NE), 5-hydroxytryptamine (5-HT), and dopamine (DA) (32–35). Acupuncture can also increase the content of brain-derived neurotrophic factor (BDNF) to prompt neurological rehabilitation (36).

### 4.1 Summary of the results

Previous investigations described acupuncture and acupressure as being effective as drug treatments, and acupressure treatments were mainly auricular points (15). Our work provides updated evidence to inspect the effect and safety of conventional acupuncture for cancer tumor-related depression. There were superior effectiveness and fewer adverse reactions to acupuncture compared to usual care. The analysis stated that acupuncture was an effective and safe therapy for tumor-related depression regardless of patients’ tumor stages or types. Acupuncture as a supplement can significantly enhance HAMD, SDS, and QLQ-C30 scores, particularly acupuncture plus usual care. The effect of electroacupuncture on HAMD scores remained to be discussed since there were only two pieces of literature.

The diagnostic criteria for tumor-related depression were different among the included studies. Three studies (16, 19, 25) applied the Chinese classification and diagnostic criteria of mental disorders (CCMD-3); the same amount of studies (17, 20, 28) used the diagnostic and statistical manual of mental disorders (DSM-5); and one study (18) utilized the international classification of disease (ICD-10). In different studies, HAMD and SDS were applied to diagnose and classify depression. The above contents may be the sources of high heterogeneity. Furthermore, heterogeneity might be correlated with various features of patients (type, status, current anti-tumor treatment, etc.) and different selective serotonin reuptake inhibitor (SSRI) antidepressants like fluoxetine hydrochloride capsule (25), sertraline hydrochloride (17, 18, 20), escitalopram oxalate tablets (18, 27), or all of the above (20). Most studies recruited manifold categories of tumor, except for two studies that involved breast cancer patients. While the subgroup analysis performed to explore whether acupuncture was more beneficial for breast cancer patients was inaccessible. Hence, we were incapable of judging the differentiation in multiple tumor types.

Due to special characteristics of acupuncture manipulation, major trials failed to blind participants and personnel. To a certain extent, this could lead to subjective consequences. Traditional Chinese medicine emphasizes treatment based on syndrome differentiation. The acupoints, duration, frequency, and course varied widely depending on individuals. Unfortunately, we were confined to conducting a subgroup analysis to determine the effect of different acupuncture regimens on tumor-related depression because few articles were available.

GV20 was the most frequently used point, in accordance with the result of metrological analysis (36), followed by GV24^+^, LR3, LI4, PC6, and HT7. Acupuncture stimulation at GV20 and GV24^+^ could prevent and treat depression by modulating the expression of multiple neurotrophic factors (37, 38). Acupuncture at LR3 and LI4 could raise the level of 5-HT and NE (39), as well as upregulate BDNF (40). LR3 and PC6 also had an advantage in regulating glial cell line-derived neurotrophic factor (GDNF) production (41). Stimulating at the PC6 acupoint inhibited the pathological state of the HPA axis in the depressive rat model (42). Acupuncture stimulation at HT7 can ameliorate depression in rat models by increasing 5-HT expression and BDNF levels (43).

Additionally, different RCTs employed different HAMDs (17 or 24 points). These were concerned with heterogeneity. Though a multitude of studies did not take the efficacy of acupuncture as an observation index, relevant depression scales were put into use, indicating that a state of depression was prevalent in tumor patients. As to safety, acupuncture is inevitable to produce adverse events on account of insertion. However, they can recover quickly without being tackled deliberately, as long as properly operated by a professional acupuncturist.

### 4.2 Other reviews

Psychological problems like depression have a great affection on the treatment of tumors. Previous studies have reported various acupuncture techniques for managing various tumor-related complications (44–47), such as pain, fatigue, insomnia, and so on. Although the conclusions indicated the effectiveness and safety of acupuncture, the effect of conventional acupuncture on tumor-related depression alone was lacking. It may be easier to understand the effect of acupuncture by concentrating exclusively on the treatment itself. We performed more rigorous inclusion criteria to screen eligible RCTs. Unlike those studies, this one demonstrated that acupuncture had a beneficial effect on tumor-related depression and improved characters’ quality of life with fewer side effects. However, in terms of improving HAMD and SDS, acupuncture was a complementary therapy, not an alternative.

### 4.3 Limitations

There were a number of deficiencies in this review. First, a variety of studies did not design a double-blind trial with sham acupuncture as a placebo control, and several studies had small sample sizes. It is universally acknowledged that various accompanying symptoms of tumors, such as pain, sleep disorders, and other complications, may aggravate depression degree; however, the included studies did not explain the impact on tumor-related depression triggered by these factors, which may have a modest effect on our work outcomes. The overall qualities of integrated studies were not extremely compelling to support our results. Second, there were merely 10 studies; we were confined to processing subgroup analysis of various types, stages, current anti-tumor treatment, etc. High heterogeneity in each data would make a difference to the accuracy of the results. Third, the publication bias analysis described via funnel plot was infeasible because smaller quantities of studies were contained. Fourth, due to the specificity of acupuncture therapy, most studies cannot completely assure the implementation of the blinding process. Depression as a mental disorder is usually assessed by self-rating scales, which could magnify the placebo effect. Thus, it is essential to conduct a more rigorous design, like using sham acupuncture, to ensure the blinding process. Last but not least, all the trials we included were conducted in China, and there may exist ethnic differences. In conclusion, the results of meta-analysis should be treated carefully, and it is crucial to adopt additional outstanding studies for further research to support the evidence.

## 5 Conclusions

Acupuncture is effective and safe to manage tumor-related depression and should be considered a complementary therapy for tumor-related depression patients. More RCTs with rigorous designs and larger sample sizes are indispensable to verifying the effect of acupuncture on depression patients diagnosed with tumors.

## Data availability statement

The original contributions presented in the study are included in the article/**Supplementary Material**. Further inquiries can be directed to the corresponding author.

## Author contributions

Conceptualization: XL and TZ. Methodology: XL and YW. Data extraction: XZ and LW. Formal analysis: XL, LW, and XZ. Writing—original draft preparation: XL. Writing—review and editing: XL and TZ. Supervision: TZ. Project administration: TZ. Funding acquisition: TZ. All authors contributed to the article and approved the submitted version.
