# Supplementary figures and images for: Acupuncture for tumor-related depression: a systematic review and meta-analysis
Source: Front Oncol. 2023 Aug 8;13:1198286. doi: 10.3389/fonc.2023.1198286 (PMC10442935; doi:10.3389/fonc.2023.1198286)

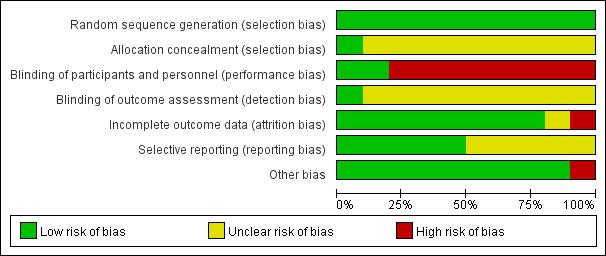

Supplement: Supplementary file 1 [file DataSheet_1.zip › Figure 2(A) Risk of bias graph.jpg]

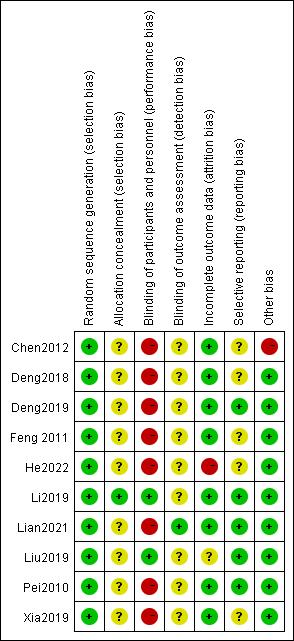

Supplement: Supplementary file 1 [file DataSheet_1.zip › Figure 2(B) Risk of bias summary.jpg]

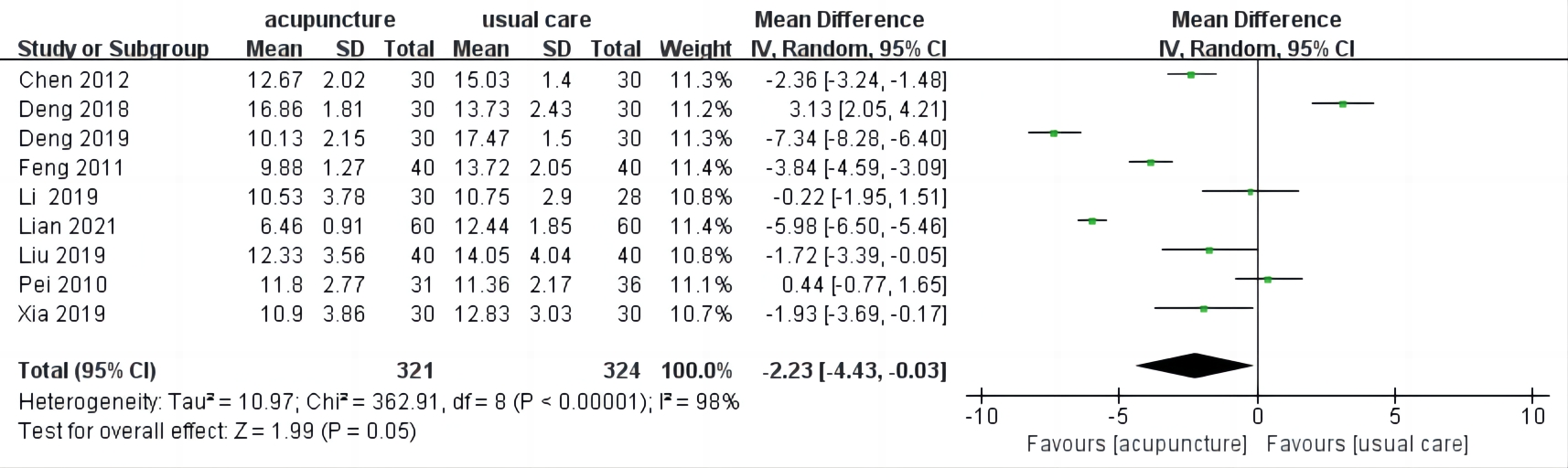

Supplement: Supplementary file 1 [file DataSheet_1.zip › Figure 3 Forest plot of acupuncture vs. usual care on HAMD.jpg]

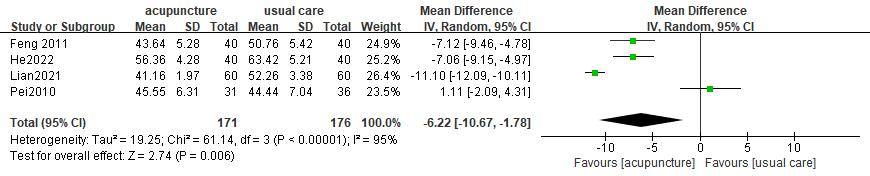

Supplement: Supplementary file 1 [file DataSheet_1.zip › Figure 4 Forest plot of acupuncture vs. usual care on SDS.jpg]

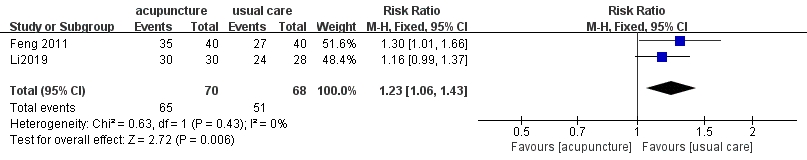

Supplement: Supplementary file 1 [file DataSheet_1.zip › Figure 5 Forest plot of acupuncture vs. usual care on effective rate.jpg]

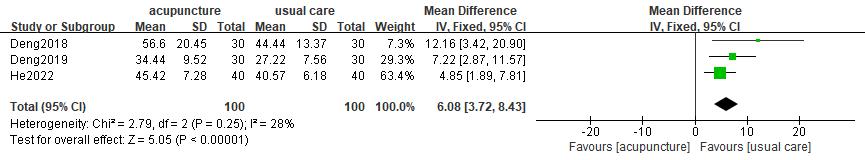

Supplement: Supplementary file 1 [file DataSheet_1.zip › Figure 6 Forest plot of acupuncture vs. usual care on QLQ-C30.jpg]

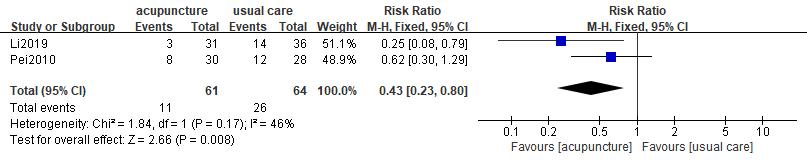

Supplement: Supplementary file 1 [file DataSheet_1.zip › Figure 7 Forest plot of acupuncture vs. usual care on adverse effects.jpg]

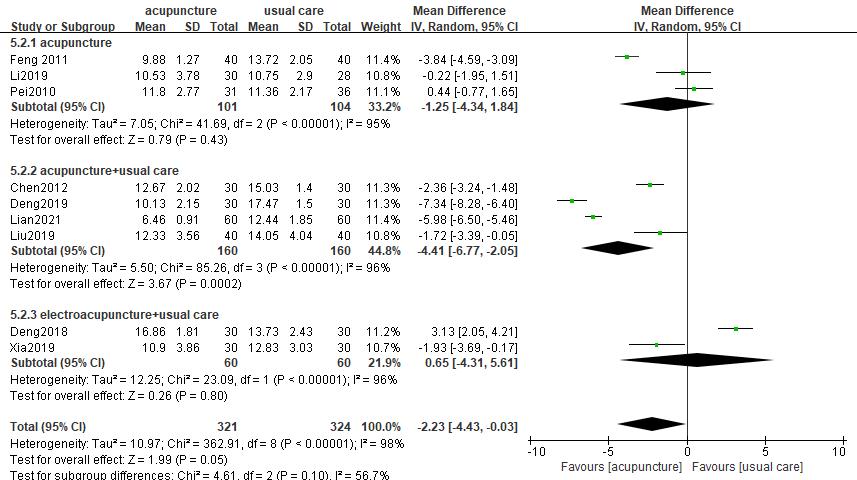

Supplement: Supplementary file 1 [file DataSheet_1.zip › Figure 8 Subgroup analysis of different interventions on HAMD.jpg]

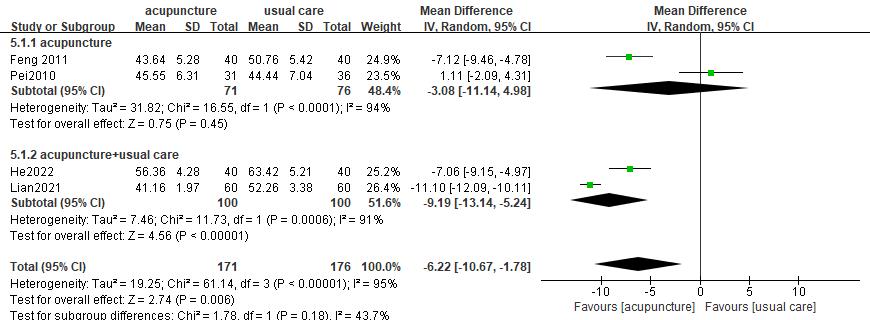

Supplement: Supplementary file 1 [file DataSheet_1.zip › Figure 9 Subgroup analysis of different interventions on SDS.jpg]
