# Supplementary material for: Acupuncture for tumor-related depression: a systematic review and meta-analysis
Source: Front Oncol. 2023 Aug 8;13:1198286. doi: 10.3389/fonc.2023.1198286 (PMC10442935; doi:10.3389/fonc.2023.1198286)
Supplement: Supplementary file 2 [file DataSheet_2.docx]

## Search strategy

**PubMed**

((((electroacupuncture) OR (needl*)) OR ("acupuncture"[Mesh])) AND (((depressive symptoms) OR (emotional depression)) OR ("depression"[Mesh]))) AND (((((((cancer) OR (benign neoplasm*)) OR (malignancy)) OR (malignant neoplasm*)) OR (neoplas*)) OR (tumor*)) OR ("neoplasms"[Mesh])) Filters: Randomized Controlled Trial

**EMBASE**

('neoplasms'/exp OR neoplasms OR 'benign neoplasm' OR 'malignant neoplasm' OR 'malignancy') AND ('acupuncture' OR 'electroacupuncture' OR 'needle') AND ('depression' OR 'depressive symptoms' OR 'emotional depression')

**Web of Science**

TS=(electroacupuncture OR needl* OR acupuncture) AND TS=(depressive symptoms OR emotional depression OR depression) AND TS=(cancer OR benign neoplasm* OR malignancy OR malignant neoplasm* OR neoplas* OR tumor* OR neoplasms)

**the Cochrane Library**

#1 MeSH descriptor: [Neoplasms] explode all trees

#2 (tumor*):ti,ab,kw OR (neoplas*):ti,ab,kw OR (malignant neoplasm*):ti,ab,kw OR (malignancy):ti,ab,kw OR (benign neoplasm*):ti,ab,kw

#3 (cancer):ti,ab,kw

#4 #1 OR #2 OR #3

#5 MeSH descriptor: [Depression] explode all trees

#6 (emotional depression):ti,ab,kw OR (depressive symptoms):ti,ab,kw

#7 #5 OR #6

#8 MeSH descriptor: [Acupuncture] explode all trees

#9 (needl*):ti,ab,kw OR (electroacupuncture):ti,ab,kw

#10 #8 OR #9

#11 #4 AND #7 AND #10

**Chinese Biomedical Literature Database (CBM)**

( "针刺"[常用字段:智能] OR "电针"[常用字段:智能] OR "针灸"[常用字段:智能] OR "针灸疗法"[常用字段:智能]) AND( "抑郁"[常用字段:智能] OR "情绪抑郁"[常用字段:智能] OR "抑郁症状"[常用字段:智能]) AND( "肿瘤"[常用字段:智能] OR "癌症"[常用字段:智能] OR "恶性肿瘤"[常用字段:智能] OR "良性肿瘤"[常用字段:智能])

**China National Knowledge Infrastructure (CNKI)**

( ( ( 主题%= 肿瘤+癌症+恶性肿瘤+良性肿瘤 or 题名%= 肿瘤+癌症+恶性肿瘤+良性肿瘤 ) AND ( 主题%= 针刺+电针+针灸+针灸疗法 or 题名%= 针刺+电针+针灸+针灸疗法 ) ) AND ( 主题%= 抑郁+情绪抑郁+抑郁症状 or 题名%= 抑郁+情绪抑郁+抑郁症状 ) )

**Wanfang Database**

主题:(肿瘤 or 癌症 or 恶性肿瘤 or 良性肿瘤) and 主题:(针刺 or 电针 or 针灸 or 针灸疗法) and主题:(抑郁 or 情绪抑郁 or 抑郁症状)

**VIP Database**

(((((题名或关键词=针刺 OR 题名或关键词=针灸) OR 题名或关键词=针法) OR 题名或关键词=电针) AND (((题名或关键词=抑郁 OR 题名或关键词=情志异常) OR 题名或关键词=抑郁症状) OR 题名或关键词=抑郁情绪)) AND (((题名或关键词=肿瘤 OR 题名或关键词=癌症) OR 题名或关键词=良性肿瘤) OR 题名或关键词=恶性肿瘤))
